# An Adaptive Game-Based Learning Strategy for Children Road Safety Education and Practice in Virtual Space

**DOI:** 10.3390/s21113661

**Published:** 2021-05-25

**Authors:** Noman Khan, Khan Muhammad, Tanveer Hussain, Mansoor Nasir, Muhammad Munsif, Ali Shariq Imran, Muhammad Sajjad

**Affiliations:** 1 Visual Analytics for Knowledge Laboratory, Department of Software, Sejong University, Seoul 143-747, Korea; nomank3797@gamil.com (N.K.); khan.muhammad@ieee.org (K.M.); 2 Digital Image Processing Laboratory, Department of Computer Science, Islamia College University Peshawar, Peshawar 25000, Pakistan; mansoornasir@icp.edu.pk (M.N.); munsif3797@gmail.com (M.M.); 3 Department of Software, Sejong University, Seoul 143-747, Korea; tanveer@sju.ac.kr; 4 Norwegian Colour and Visual Computing Laboratory, Department of Computer Science (IDI), Norwegian University of Science and Technology (NTNU), 2815 Gjøvik, Norway; ali.imran@ntnu.no

**Keywords:** education, human–computer interaction, road safety, sensor, technology, virtual reality

## Abstract

Virtual reality (VR) has been widely used as a tool to assist people by letting them learn and simulate situations that are too dangerous and risky to practice in real life, and one of these is road safety training for children. Traditional video- and presentation-based road safety training has average output results as it lacks physical practice and the involvement of children during training, without any practical testing examination to check the learned abilities of a child before their exposure to real-world environments. Therefore, in this paper, we propose a 3D realistic open-ended VR and Kinect sensor-based training setup using the Unity game engine, wherein children are educated and involved in road safety exercises. The proposed system applies the concepts of VR in a game-like setting to let the children learn about traffic rules and practice them in their homes without any risk of being exposed to the outside environment. Thus, with our interactive and immersive training environment, we aim to minimize road accidents involving children and contribute to the generic domain of healthcare. Furthermore, the proposed framework evaluates the overall performance of the students in a virtual environment (VE) to develop their road-awareness skills. To ensure safety, the proposed system has an extra examination layer for children’s abilities evaluation, whereby a child is considered fit for real-world practice in cases where they fulfil certain criteria by achieving set scores. To show the robustness and stability of the proposed system, we conduct four types of subjective activities by involving a group of ten students with average grades in their classes. The experimental results show the positive effect of the proposed system in improving the road crossing behavior of the children.

## 1. Introduction

Technology has impacted almost every aspect of life today and has dramatically expanded access to education [1]. Nowadays, sensors can measure one’s pose, facial expression, blood pressure, energy consumption, and physical awareness [2,3]. Smart simulators utilizing such sensors can be built to educate and train children in a safe environment. There are application domains wherein education in the physical world can be impossible or undesirable, such as road safety. Children’s road safety skills are not yet fully developed, and they also lack knowledge and experience about how to behave correctly in different traffic situations [4,5]. Therefore, effective road safety education is vital for children, and must include teaching theoretical knowledge, as well as a fair amount of practical exercises [6].

Children’s road safety training in real-world environments is associated with several difficulties. Letting children practice in real traffic environments exposes them to potential danger, and therefore, prerequisites must be fulfilled before practical exposure to the real road [7]. Furthermore, traffic density and speed need to be significantly reduced at the time and place of training, and the children must be accompanied by a specialized person who can properly organize and overlook the whole procedure. This makes it harder for educational institutions to provide enough practical traffic safety training to their students. In order to deal with these problems, VR-based training seems a promising solution [8]. In VR, the real-world environment is substituted by a realistic and safe VE. Previous studies have confirmed the enormous potential of this approach [9,10]. VR has the potential to facilitate knowledge acquisition, content understanding, effective communication, and problem-solving skills [11,12,13]. The implementation of a completely functional VE facilitates learning among people of various ages, and is safer than real-world experience [14]. VR is also cost-efficient and capable of reducing potential risks [15,16,17]. 

Virtual systems are used in different areas for training purposes, such as medical device use, military training, and traffic simulation [18,19,20]. Children’s road safety is a top priority, as they lack education and awareness related to road crossing and other safety measures [21]. Child injury rates are four times higher than in adults [22,23]. However, the VR systems discussed in the literature served mainly as simulators, while the tutoring task was carried out by human tutors. Moreover, these systems were limited to specific aspects of traffic safety and offered a fixed set of exercises that were the same for every user. The existing research in this direction has several drawbacks, such as the lack of interest of the students in theoretical learning, that result in poor education. These setups are expensive and require complex system configuration. These training systems are not appropriate for small children, as they use head-mounted display devices that may cause dizziness, headaches, and nausea. Similarly, handheld controllers create problems during interaction with the VE because of their tracking limitations [24]. The VR training systems presented in the literature involve minimal user interaction with the VE. We believe that increasing flexibility while decreasing human dependency would greatly benefit the broader dissemination of VR training. 

To educate children about road crossing, we propose a VR-based training system. The proposed system offers an open environment, a wide range of exercises and different interface setups, as well as a child performance evaluation system (CPES) that tells us about the performance of a child during road crossing in VE. Our system consists of three main components: (a) interface (immersive, semi, and non-immersive), (b) city simulation, and (c) CPES. In the road safety training system, we used a traditional display mode that supports both single-screen and multiple-screen displays. It is a viable solution to be implemented in an institute and at home and is suitable for a trainer to monitor the user’s activities in a VE. The proposed system aims to educate children to follow road signs properly, cross road intersections and junctions carefully at designated zebra crossings, observe traffic lights, and walk properly on the pedestrian walkways. In the training system, we modeled a 3D realistic virtual city with virtual intelligent traffic systems. In the proposed system, the trainee can control the avatar in the VE through his/her body movements, creating the illusion that the trainee is walking around in the environment. We developed a virtual city with an intelligent traffic system in which all the vehicles and pedestrians are intelligibly trained. A virtual avatar is a special player in the VE that represents the trainee, enabling them to move around in the road safety training system. Instead of handheld controllers and head-mounted devices, a keyboard, mouse, and Kinect can be used for virtual avatar control. Kinect is an inexpensive device, and it can be used easily with computer systems. It is a motion-sensing input device that takes human body gestures as input data. It allows the users to interact with the VE without the need of a game controller. It enables the user to move and interact through a natural user interface using gestures and spoken commands. To interact with the environment, the trainee must move in front of the Kinect’s field of range. The virtual platform uses a Kinect sensor to receive the gesture input. It then transfers this gesture into an interaction within the VE. The software requirements and the tools that are used in designing the VR-based road safety training system are Unity, Microsoft’s Kinect sensor, Visual Studio for programming in C# language, Adobe Fuse, and Autodesk Maya for modeling the characters and the environment. The main contributions of the paper can be identified as follows:

- The 2D video- and classroom-based training approaches are less helpful for children, as they get bored of continuous learning. In this paper, we employ Kinect in a 3D interactive and immersive environment to provide a safer way of children’s training. To deal with students, especially children, visual and physical interactions play a vital role, as children get attached easily when they are involved as subjects themselves in any activity. The present study explores the hidden potential of a 3D VE to educate children about road safety skills in an interactive manner, with their safety on the top priority;
- Most children are interested in participation rather than watching continuous lectures and activities. They concentrate closely on interactive response learning activities. Students may get bored with the traditional learning processes that are used in our educational institutes. To make the learning process enjoyable, we developed a 3D interactive and immersive road safety game, which stimulates the cognitive learning abilities of children and trains them in an interesting way;
- Instead of using handheld controllers, we implement a Kinect in our system, connected to an immersive digital game for receiving gestures as the input in a 3D VE. The Kinect data reflect reality in VE in a specific way that attracts the students towards learning road crossing rules. The experimental results show the interest of children in obeying traffic rules and we observe a positive effect on their learning rate and awareness;
- Institutions with limited resources (both time and financial) can easily afford the presented training system, and the proposed interactive and virtual application can be used to train several students in a limited time. Similarly, a single Kinect device along with its setup can be used to train a whole class, which is an economical yet effective solution for a child training.

The remaining paper has four sections. In Section 2, the VR literature is investigated, highlighting the current achievements and limitations of the existing systems. The proposed methodology along with its detailed working mechanism is explained in Section 3. Experimental results, simulation outputs, and subjective analyses are given in Section 4. We finally put forward our conclusions and prospects for future development in Section 5. 

## 2. Related Work

Many studies have shown the positive effects of immersive and interactive learning as compared to traditional methods of learning in a 2D environment, such as books, videos, and PowerPoint presentations. Technology and advanced interactive algorithms have performed well in many data science problems [25,26,27]. Therefore, researchers recommend VR games and simulators for learning and polishing training skills [28]. In the following sub-sections, the literature about gamification and virtual training is covered. 

### 2.1. Gamification

Gamification is a method of building the interest and motivation of users toward non-game systems by incorporating elements of game design. The gamification concept consists of eight elements, including rewards, stages, leaderboards, challenges, avatars, progress bars, enjoyable voices, and feedback. However, rewards, leaderboards, and challenges have higher potential in attracting the attention of participants [29,30]. Besides this, the rate of attraction and motivation toward a gamified environment depends on the habits of users and the tasks being handled [31]. Combining the concept of gamification with a VR system can improve the rate of attraction and engagement of users in non-game-context activities. VR systems can be divided into three categories, including non-immersive, semi-immersive, and fully immersive setups. The immersive setup of VR provides a real environment-like experience to users that enables them to interact with virtual contents by performing certain tasks [32]. Gamified VE attracts the attention of a participant more effectively than a game alone due to the addition of immersion with gamification [33]. Further, the incorporation of challenges, avatars, and feedback in the form of rewards or points as gamification elements into VR-based games facilitates a higher rate of user attraction and motivation [31]. Thus, a high-level immersive experience with gamification is stimulating, and users try to complete challenges with greater attention, engagement, and motivation.

### 2.2. Virtual Environments

Many studies have shown the positive effect of VE in various types of training, such as healthcare [34], tourism [35], and education [36,37]. Maiano et al. [38] proposed a virtual situation composed of potentially harmful content that can cause real stress. Since a student better absorbs a lesson under dangerous conditions and in VE, the same reasoning is applied. In a VR-based 3D environment, imitative learning can also be achieved, as we assume that the acts and movements of a 3D virtual player are copied by students. There is a bond between the students and the player in the VE, because the students interact with the player. The same relationship was established by Fox et al. [39], in which students stood in front of a 3D avatar that was eating. The avatar was trained to gain body weight when it ate chocolates, and when it ate carrots its weight reduced. However, when it ate something other than carrots and chocolates, its weight remained the same. At the end of the immersion activity, the student performed an activity in which they controlled the avatar in VE and followed the behavior of the avatar that they had noticed in the previous activity. Female students make small use of chocolates, while boys made use of a lot of chocolates. When the emotions of the students are involved, there are positive effects on imitative learning [40]. Milgram et al. [41] performed an activity in which a student foisted another student with an electric shock. They controlled two avatars in a VE through fake electric shocks. Their activity was under the control of a virtual physician. The virtual avatars had the ability to feel pain. Slater et al. [42] noticed that the subjects’ reactions matched. Thus, a similar line of conduct in this instance can be expected from the subjects. 

### 2.3. Virtual Environments for Road Safety Education

VR-based gamification in the context of road safety has the potential to improve the knowledge and recognition of hazardous situations, and the way to handle these situations properly by providing safe and immersive environments wherein users can practice activities on the road in different dangerous situations [43]. Several studies have been conducted in this context; for example, Zare et al. [44] designed an interactive visual system to improve the street crossing behavior of school children in the presence of parents. They confirmed the improvement of street crossing knowledge in children after participation. However, they did not use immersive setups and considered only street-crossing behaviors. Further, Jiang et al. [45] proposed a gamified immersive setup in which they analyzed the behavior of children during road crossing with their fellows. They classified the children’s behaviors into risky and safe categories based on the gap between vehicles and children’s avatars. The study showed how children interact with computer-generated agents while crossing the road using an immersive setup. However, the limitation of their framework was that they did not analyze the behavior of participants before and after the participation in terms of safe road crossing. To analyze the behavior of pedestrians, Jiang et al. [46] proposed a VR-based framework in which two experiments were conducted, one in which the user crosses roads with a programmed computer-generated agent, while in the second experiment, they allowed users to cross the road in a virtual environment without following the agent. They found that users who had experiences with risky agents were in more danger, and participants who had experience with safe agents were in the safe category. Furthermore, their results confirmed the huge influence of the VR-based system in the context of road crossing, but their framework used only manual devices, such as a mouse and keyboard to control the avatars that were the source of the engagement and motivation of users when using and learning from the VR system. 

In France, there is a road safety and risk awareness campaign called “Permis Piéton”. The campaign aims to foster children’s attentiveness to hazards on roads. This campaign was launched in French classes and on the Internet, there are many small games for children’s awareness. However, none of these games belong to the 3D immersive category, and children cannot participate physically. At an early age, students have no proper knowledge of road crossing techniques [47,48]. Charron et al. [49] stated in his work that training children in immersive environments can increase children’s learning skills and they become able to avoid risks while using the road. McComas et al. [9] reported in a study with fourth- to sixth-grade students attending urban and suburban schools. Through a VR intervention, the participants were supposed to learn several pedestrian safety behaviors. In their activities, children showed a significant improvement within the VR application, and those from the suburban school transferred their improved behavior into real-world behavior. Thomson et al. [23] studied different techniques for selecting the right ways to cross a road. The study participants were 7-, 9-, and 11-year-old students, and the training was conducted using a VR system that lets the user examine traffic and decide at the moment to initiate crossing. Their results were positive, students were able to estimate their crossing times better and showed improvements in finding safe opportunities to cross roads after training in VR. Schwebel et al. [50] proved the validity of using a specific VR system as a tool to understand and prevent child injuries while using the road. The user could initiate crossing by either shouting or taking two steps forward in the virtual system. The actual crossing was then performed automatically without any further action by the user. The outcome of their study indicated that behavior in the real world matched the behavior in their VR system. Schwebel et al. [51] also studied a similar Internet-based VR system. Some other immersive systems have also been developed in which students can walk through virtual 3D environments [52,53]. 

To create more interest and motivation in users and especially in school children, towards safe road crossing, researchers should integrate various devices that allow the control of the avatar in the VE by physically performing the same action in the physical world, which involves a more immersive setup, such as using stereoscopic screens and incorporating more gamification elements into the system. In our road safety training system, we have used a traditional display mode that supports both single-screen and multiple-screen displays. It is a viable solution to implement in the institute or home, and is suitable for a trainer to monitor the user’s activities in a VE. In summary, it can be concluded that the studies mentioned above all demonstrate the enormous potential of VR for children pedestrians’ safety. We believe that increasing flexibility while decreasing dependency on human assistants would greatly benefit the broader dissemination of VR training. Our proposed road safety training system presented in this paper builds on the success of these studies and aims to bring VR training one step closer to actual use in schools and kindergartens by using state-of-the-art technology from the entertainment industry and research in technology-enhanced learning. The next section presents the proposed system and how these principles have been implemented in the software.

## 3. Methodology

The most robust type of developed environments for creating rich virtual worlds and games are called game engines [54,55]. Some of the widely used game engines include Unreal Engine, Unity, and CryEngine. The choice of which one to use depends on what we would like to achieve and what integration options with hardware sensors are present. We picked Unity as the game engine because of its simplicity, quick prototyping abilities, and existing Kinect plug-ins. The game engine itself is written in the C/C++ programming language. On the other hand, C# can be used for writing and executing game-logic scripts with Unity. Different sensors on the market can detect natural human gestures and behavior, which can be used to develop human–computer interaction applications. Amongst the most used sensors are the ASUS Xtion and the Microsoft Xbox 360 Kinect sensors. Both these devices are equipped with an RGB color camera and an infrared/depth sensor. The ASUS Xtion offers better RGB image quality without any external power supply.

The driver’s quality of this sensor is lower compared to the Kinect, and the sensor has no motor, thus is unable to re-position itself in real-time. Kinect, on the other hand, has better driver support, 2 degrees of freedom for its motor, and can rotate along its vertical axis. For our educational platform, we chose the Kinect sensor over Xtion since we wanted to track human skeletons and gestures. Skeletal and gesture tracking has two requirements, i.e., the quality of the depth sensor and the ability to re-position the tracking device. The depth sensors on both devices are of equal quality. That is why the decisive factor was the Kinect’s ability to reposition itself in real-time without manual positioning by a human. Furthermore, Kinect is a more popular device among developers, and it is easier to integrate with Unity.

**Figure 1:**
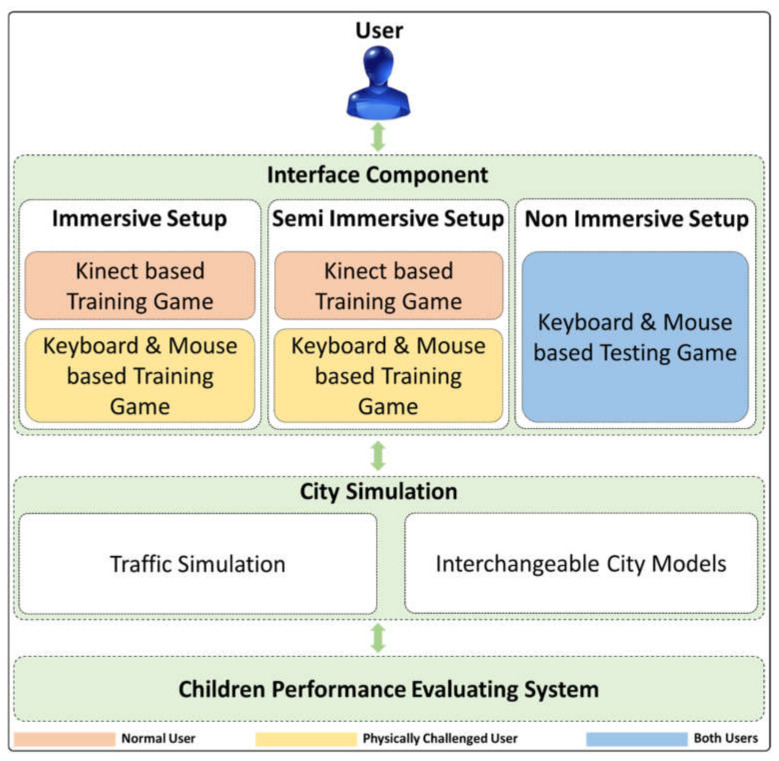
Our proposed system architecture consists of three major components. The interface component connects the user to the city simulation and supports different hardware setups, varying in degree of immersion, availability, and cost. The city simulation including traffic and urban neighborhood is the core of the architecture and generates the virtual training environment. Additionally, the city simulation is also connected to the CPES. Furthermore, physically challenged students can control walking, jump, and running gestures with the help of a keyboard.

**Figure 2:**
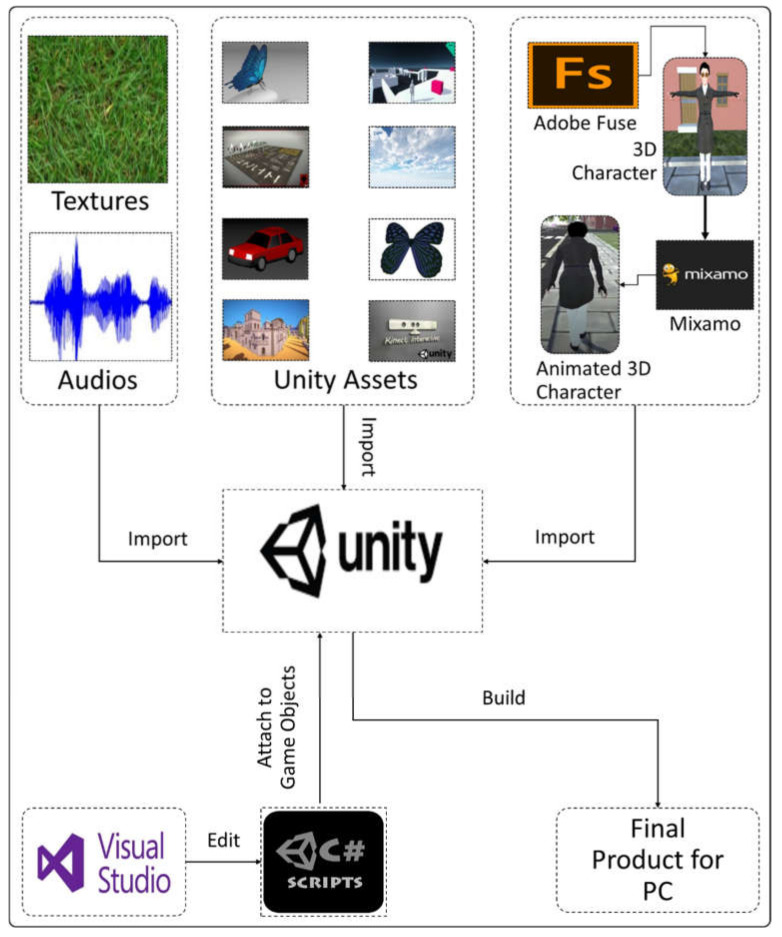
The development flow of our proposed system, wherein we picked Unity as the game engine with Unity assets, third-party 3D models, audios, textures, and existing Kinect plug-ins downloaded in compatible formats and imported into Unity. The 3D character models are designed in Adobe Fuse and animated with the Maximo third-party application. C# is used as the programming tool for logic implementation.

**Figure 3:**
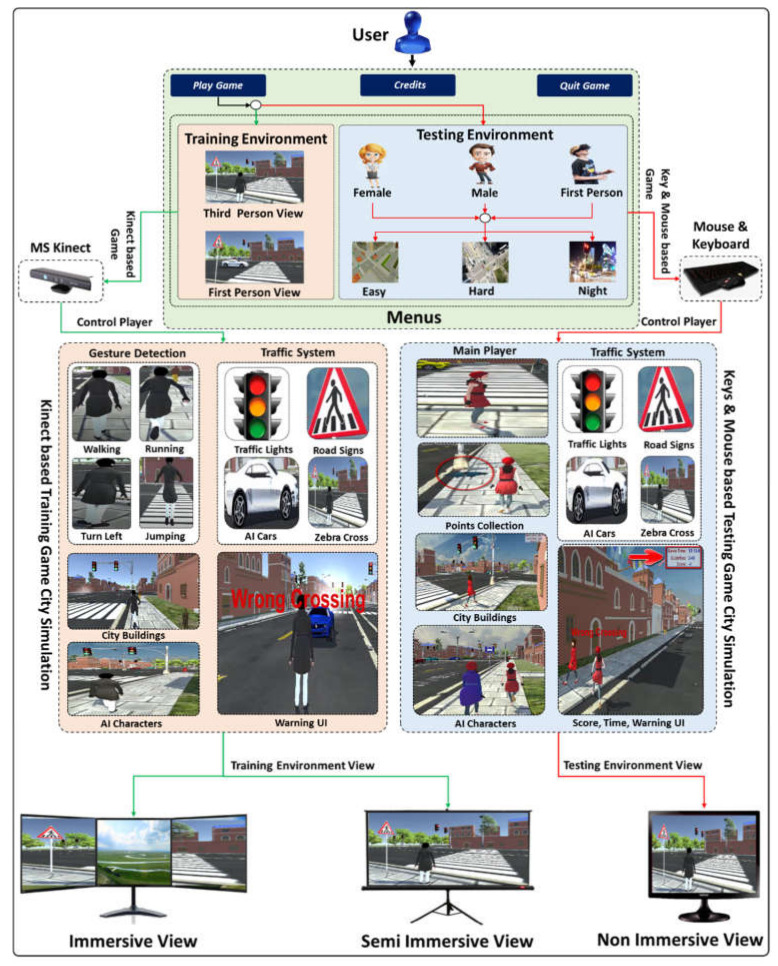
There is a training and a testing environment in the output screen of our application and a user can choose anyone of them. The training environment uses Kinect as the input device and the testing environment has a keyboard and mouse for interaction. The proposed system supports different hardware setups, varying in degree of immersion, availability, and cost.

The following describes the concept of creating a VRBTS that is highly flexible and mostly independent of human trainers. The proposed VR training system offers an open environment, a wide range of exercises, different interface setups, as well as a CPES that tells us about the performance of the children in VRBTS. This section will first present the overall architecture of the system and subsequently describe the individual components. Our proposed VRBTS’s architecture consists of three significant components. (a) The interface component connects the user to the city simulation and supports different hardware setups, varying in degree of immersion, availability, and cost. (b) The city simulation, including traffic and the urban neighborhood is the core of the architecture and generates the virtual training environment. (c) Additionally, the city simulation is also connected to the CPES. The CPES analyzes information from the city simulation and adapts the training to the individual needs of the user. Figure 1 shows the overall architecture of the proposed system. Figure 2 shows the development environment, and Figure 3 shows the overall application interface.

In the first phase, we designed the complete environment, with main players, AI cars, pedestrians, buildings, roads, trees, etc. For character modeling we used Adobe Fuse software. Adobe Fuse is a 3D computer graphics software introduced by Mixamo and used to create 3D characters. Mixamo is also used for character rigging and animation. We used different packages from the Unity assets store for designing buildings, roads, traffic lights, cars, road signs, and environment beautification. For the AI involved in the cars, traffic lights and pedestrian behavior, and all the system management processes, we used the C# programming language. Unity has the built-in support of Visual Studio IDE, which is used for editing C# scripts. Unity was the main workstation of our project. All the development aspects, including characters and scripts were integrated into this phase and an intelligent system was built.

### 3.1. Interface Component

**Figure 4:**
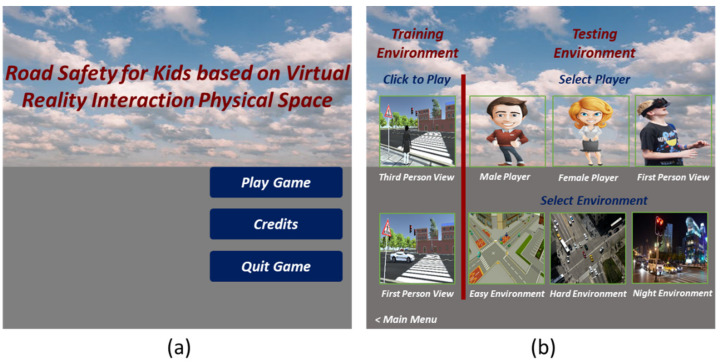
The interface of the VRBTS, where (**a**) represents main menu, while (**b**) shows the players and environments selection menu.

With the advances of the digital entertainment industry, a wide range of display and input devices have come into the market that can be used for interactive real-time 3D applications to achieve different levels of immersion. A high level of immersion leads to more natural behavior, and thus promises better learning results. However, due to temporal, spatial, and budget restrictions, the requirements of a highly immersive interface cannot always be fulfilled. Therefore, our VRBTS supports different kinds of interface setups. The proposed VRBTS consists of different buttons, such as Play Game, Quit Game, Credits, Third Person View, First Person View in Training Environment, Male Player, Female Player, First Person View, Easy Environment, Hard Environment and Night Environment, and Main Menu in Training Environment (Figure 4a,b). The VRBTS offers multiple levels, wherein a level is a route that needs to be followed by the avatar that is controlled through student body movements, abiding laws and regulations at the same time. It has a start, where the user chooses an avatar (male, female, or first-person view). This is the initial position of the avatar in the 3D VE. The avatar collects butterflies as it progresses through the game.

#### 3.1.1. Immersive Setup

**Figure 5:**
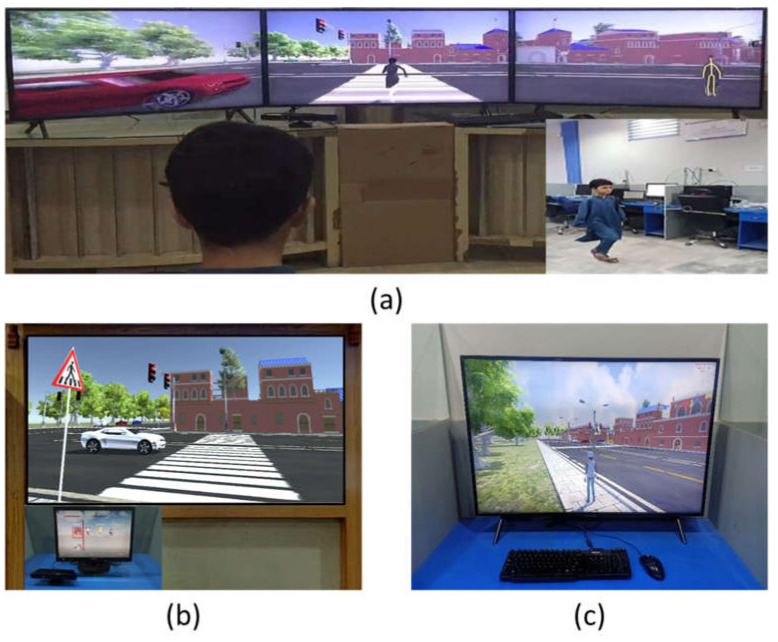
Different display setups of VRBTS where (**a**) shows immersive setup, (**b**) represents living room setup, and (**c**) is a desktop setup.

The display component of the immersive setup in our VRBTS consisted of three active stereoscopic monitors arranged in a semi-circle with the following specifications: FY3D-V-55 model number, 50-inch monitor size, and 4k resolution that provides a 3D experience to users with the naked eyes and enables a 3D view into the virtual world at a visual angle of 180 degrees, as shown in Figure 5a. As the interaction device, the Xbox 360 Kinect Sensor was used, which performs full body tracking of the user. A powerful computer with high-end video hardware is required to run this setup. It is thus still considerably more costly than the other setups listed below. 

#### 3.1.2. Living Room Setup

In the living room setup, the three active stereoscopic monitors were replaced with a multimedia projector developed by Optoma, model GT1080HDR, with characteristics of 1080p resolution, 50K:1 contrast ratio, 4k input support with HDMI, VGA and USB-A, that are generally ideal for gaming activities. The display surface, Sable frame B2 series, gives a 16:9 aspect ratio and can offer up to ultra-HD experiences to participants. The Xbox 360 Kinect Sensor facilitated the required interaction, as in the previous setup. Although the display component here was less immersive compared to the setup mentioned above, but it still facilitated natural interaction. This setup fits in an average living room and a normal computer is enough to run it. Since most components of this setup can be found in an average household, it only requires minor additional costs to be used in a home setting. Figure 5b shows the living room setup of the proposed system.

#### 3.1.3. Desktop Setup

The desktop setup uses a standard monitor manufactured by Samsung with specifications of a 43-inch screen size, 4K resolution, and HDMI and USB connectivity, and it can support auto game mode as a display with a keyboard and mouse as the interaction devices, as shown in Figure 5c. The degree of immersion allowed by this device is inferior compared to the other setups in terms of visual representation and interaction. Furthermore, a mouse and keyboard are used to interact with the system instead of body gestures. As most computers have these standard input devices, no additional overheads are required to run the VRBTS in this setup. Additionally, since notebooks have built-in keyboards and pointing devices, the mobility of this setup is superior. Furthermore, the VRBTS can run in this setup as a standalone application. In summary, the desktop setup is a lightweight alternative for cases when the immersive or living room setup are not available due to cost.

**Figure 6:**
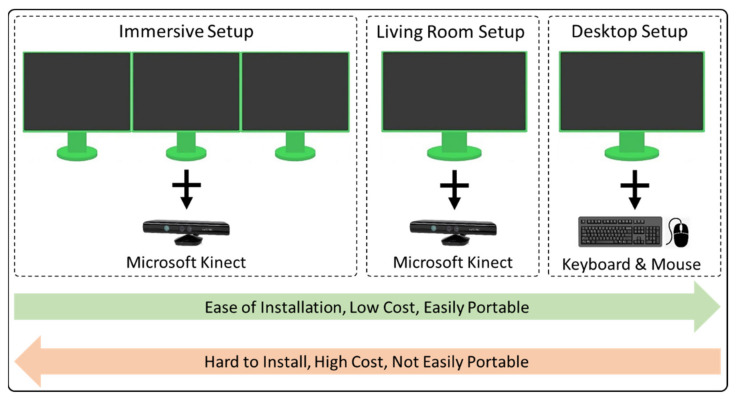
Different VR training system interface setups.

To conclude, the VR training system can support three interface setups that differ in terms of requirements and degree of immersion. Figure 6 illustrates the different setups next to each other, while Table 1 offers a comparative analysis of the abovementioned setups of our proposed system.

### 3.2. City Simulation

**Figure 7:**
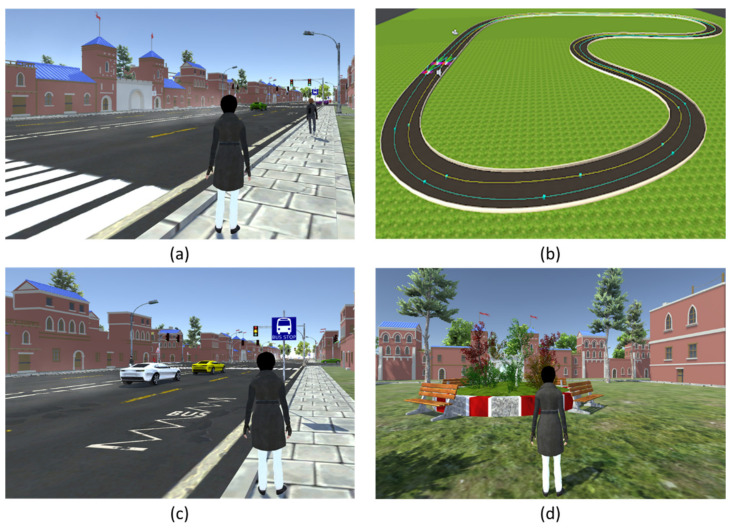
(**a**) represents the virtual city, (**b**) shows the waypoint system, (**c**) depicts cars, walkways, bus stops, and road signs, and (**d**) shows a park in our simulated city.

The city simulation was developed using Unity and consisted of a multi-agent traffic simulation as well as realistic 3D models of urban architecture, as depicted in Figure 7a. A waypoint system is placed along the roads of the virtual city to direct and control traffic, as shown in Figure 7b. Cars were generated at dedicated waypoints with a certain degree of randomness in terms of the waiting period, type of car, and speed. Due to the randomness, each time the simulation is started, the user will encounter different traffic situations, just as in the real world. Each vehicle works as an artificial intelligence agent. The default behavior of the agent is to simply get from one waypoint to the next. The vehicles were deigned to react to traffic infrastructure, such as traffic lights and zebra crossings, adapt their speed if the car in front is slower, and choose at random between different subsequent waypoints if there are multiple waypoints connected to its current goal. The movement of the cars is based on the physics simulation of Unity to achieve realistic acceleration and braking behaviors. The traffic simulation is not restricted to work with only one urban environment, and can be adapted to different environments and road networks. Therefore, the appearance of the virtual training environment can be altered by changing the 3D models. 

For this project, we utilized third-party models of stereotypical urban architecture to create virtual cities. Using a real-world city increases the degree of realism and allows children to practice routes from everyday life, such as their actual route to school. The environment created for this simulation consists of urban buildings, houses, roads, moving cars, walkways, traffic lights, bus stops, road signs, a kindergarten, parks, and user-controlled avatars, as illustrated in Figure 7c,d.

There are several subsystems, developed on top of our VE, that help and assess user interactions. Those subsystems are described below.

#### 3.2.1. Street Traffic Subsystem

This part of the simulator governs the spawning locations of cars on the road, and the number of total moving vehicles in the VE (we need to keep them to a minimum, due to the complexity of the path-planning and path-finding algorithms). It also controls the traffic lights. In the traffic subsystem, we have traffic lights for vehicle control. These traffic lights are the same as real-world traffic lights. All the vehicles follow these traffic lights while patrolling in the VR environment. We used road signs for the guidance of children at particular locations, such as bus stops, school stops, zebra cross, road turns, etc. Furthermore, streetlights were incorporated for the night version of the game.

#### 3.2.2. Vehicle Artificial Intelligence

This subsystem steers the vehicles clear of any obstacles, and makes them aware of the surrounding environment. Artificial intelligence makes the moving car stop if there is an avatar in front of it, a pathway with a vehicle crossing it, or a red traffic light. AI also determines the driver behavior. As a result, the system gives a warning on the screen and alerts the user. 

#### 3.2.3. Avatar Gestures

Five different gestures are implemented in the VRBTS. With the help of these gestures, the trainee can move the virtual avatar in the 3D VE.

##### Walking and Running

**Figure 8:**
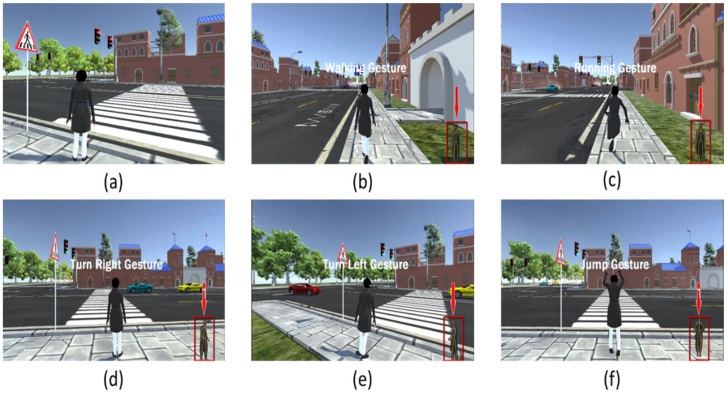
Different gestures of the main avatar: (**a**) idle, (**b**) walking, (**c**) running, (**d**) turning right, (**e**) turning left, and (**f**) jumping.

When the game is played, the avatar is in the idle position with an idle gesture (Figure 8a). After that, when the player starts moving in front of the Kinect sensor, two different gestures are implemented, one for walking and the other one for running. The student or trainee moves the avatar forward into the VE by the movement of his or her knees. The walking gesture is defined by moving both knees in a forward direction on a fixed position toward the Kinect in a sequence, while the running gesture involves both knees moving in an upward direction. Physically challenged students can control the walking and running gesture with the help of the arrow key buttons on the keyboard in the VE. The controls for physically challenged students are as follows: The in-game menu provides an option to “lock” the Kinect sensor controlling the avatar. That way, anyone in a wheelchair can still use the platform by using the directional keys on the keyboard. There is also a semi-lock mode, in which the Kinect sensor controls the avatar’s head rotation, and the avatar’s movement is handled via the keyboard. Figure 8b,c shows the walking and running gestures of the main avatar.

##### Turning Left and Right

Two other different gestures are implemented in the VRBTS, one for turning left and the other one for turning right. The turning of the avatar is achieved by moving the relative orientations of the shoulders towards the Kinect sensor. If the right shoulder is further away from the sensor than the left one, the avatar turns right, and vice versa. Figure 8d,e shows these gestures of the avatar.

##### Jumping Gesture

The jumping gesture has been implemented in VRBTS. This gesture is detected when the student jumps high enough in front of the Kinect sensor. Physically challenged students can control the jumping gestures with the help of the spacebar button on the keyboard. The jumping gesture is shown in Figure 8f.

#### 3.2.4. Avatar Movement 

There are three setups in the proposed road safety training, i.e., immersive, semi-immersive, and non-immersive. In immersive and semi-immersive setups, the avatar movement is controlled by the Kinect sensor. In the case of physically challenged students, as they cannot move in front the Kinect, they can use the mouse to determine the direction of the avatar, while for running they use “up arrow”, walking is “down arrow”, turning right is “right arrow”, turning left is “left arrow”, and they jump with the “space” button on the keyboard. Similarly, in the case of a non-immersive setup, all the movements of an avatar, such as walking, running, etc., are controlled by keyboard keys, while its direction is handled by the mouse.

### 3.3. Children Performance Evaluating System

**Figure 9:**
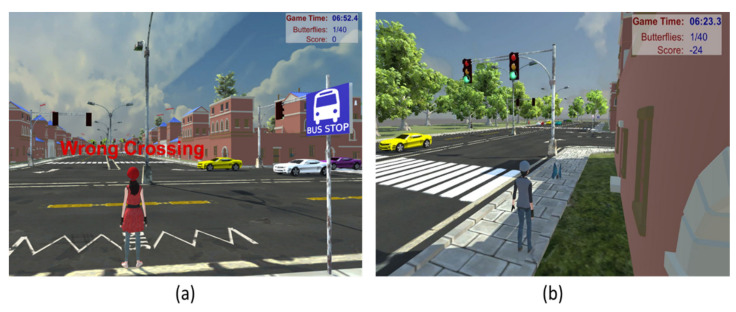
Children performance evaluating system where (**a**) shows the warning UI system and (**b**) represents the scoring system.

CPES offers a set of different exercises for various traffic situations. CPES is employed to adapt these exercises automatically in a way that should optimize learning results. Furthermore, the system gives feedback on the child’s position and movements. It is a visual layer that shows real-time statistics, such as elapsed time since the start of a level, the current number of collected butterflies, the total number of butterflies to be collected, warnings to the trainee, and total score. Finally, the platform evaluates the overall performance and grades the child (Figure 9a,b). Avatar actions that follow the rules, regulations, ethical practices, and state law requirements are awarded a positive reward. On the other hand, actions that are wrong or could lead to an injury in the real world are discouraged and sanctioned, and points are taken away. The maximum number of butterflies that can be collected at a level is 40. After collecting all 40 butterflies, a large butterfly appears, and after catching the last butterfly, a summary screen appears, showing how well the child has performed.

#### Audible and Visible Alarm System

**Figure 10:**
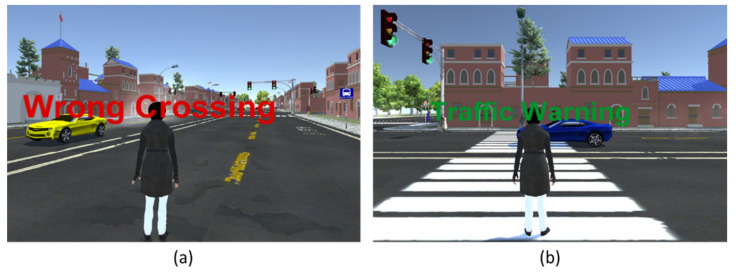
Audible and visible alarm systems where (**a**) shows wrong crossing on the road and (**b**) represents wrong walking on a zebra cross.

One essential function of the environment is to alert the player when they incorrectly cross the road. Both audio and visual alarms are used (Figure 10a,b). When a student is walking on the road in the VE, a red-colored text appears on the screen along with audio to alert the user. As a result, a player moves to a safe location immediately.

## 4. Experimentation

Students in primary- and middle-level education are the targets of our virtual simulation, but the system can be used by students of any age. The focus of our system is children, as their voluntary skills are not yet fully developed.

**Figure 11:**
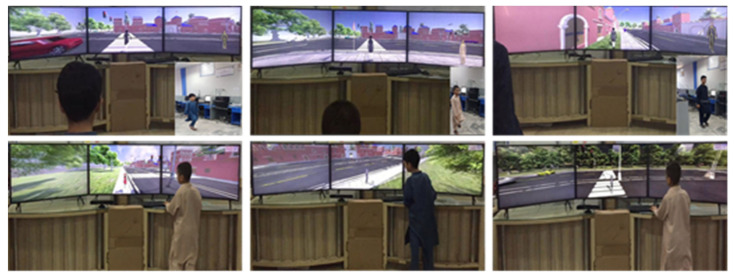
Students performing training activities.

The simulator’s primary purpose is to build voluntary and involuntary habits in young children, over a wide range of situations, such as correctly crossing a street on the pathway, following traffic light rules, learning and following road signs, safely walking on the sidewalk and correctly waiting for the bus at a stop. In our VR training system, a Kinect sensor detects and recognizes the gesture input of a student and then transfers it to VE for effective interaction. There are two types of interactions, the directional movement of a 3D avatar (gestures) and manipulation of the in-game menu. Different types of activities were performed by a group of 10 students in a local city school to demonstrate the stability and robustness of our proposed system. We studied the responses and improvement level of the students after performing different activities in the learning and testing sessions. Children with average grades in their classes at ages 8 to 15 participated in the subjective evaluation. These children were selected by their teachers for participation in the activities (training, testing). These activities were performed in the presence of their parents and teachers. In Figure 11, we show visuals from our activities. Details of different learning and testing sessions are discussed in the subsequent sections.

### 4.1. Testing Children’s Knowledge before Training (A-1)

**Figure 12:**
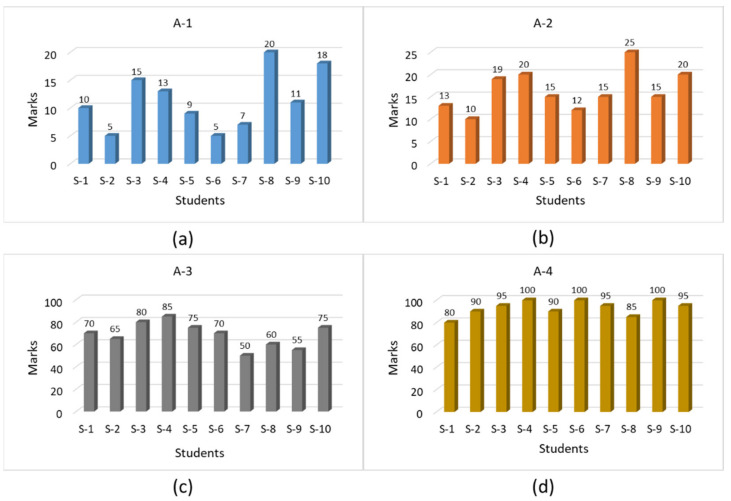
(**a**) test score before training, (**b**) score after traditional methods-based training, (**c**) score after VR-based training, (**d**) score after self-practice and children’ gaming.

Before starting the learning activity, we first tested the student’s knowledge about road signs and safety measures, focusing on the related skills of pedestrian behavior. This test was paper and pen-based with questions about road signs, traffic lights, road crossing, different stops on the roadside, and other things. We checked the results of each student to save the record for later analysis. Figure 12a shows the record of the students in this test session.

### 4.2. Traditional Methods-Based Training (A-2)

In this session, we performed the learning activity for road safety skills. This activity was based on the video demos, graphs, models, and charts. After a 40 min learning session, students performed the same test that was conducted at the start of the activity (A-1). The results of this testing activity (A-2) are shown in Figure 12b. During this session of the training, children were very bored, and a few of them slept in their chairs during the activity. However, the results were improved a little, but this was not our target. Students were refreshed after this session and then we started the next session.

### 4.3. VR Based Training (A-3)

This training activity (A-3) was performed on our VR-based road safety training system. We studied the efficiency of our system from a functional and cognitive point of view. Before starting the actual training, a 15 min demonstration video was presented to the students, explaining how to use and interact with the system and how to control and interact with the available interfaces. When the children had learned about the system completely, they started exploring the roads of the VE by themselves. At the start, they were just enjoying the environment, walking on the roads without noticing traffic lights and being hit by vehicles again and again. However, when they properly understood the concept of the game, the warnings on wrong crossings, and the motivation of getting higher scores, they became serious and started properly crossing the roads by waiting for the traffic signals. This session was about two hours long and the students were just enjoying and playing. The total time for an individual student was 10 min each with ±3 minutes’ setup and exchange time excluded. At the end of the session, they took the test, and their results are shown in Figure 12c.

### 4.4. Children’s Gaming and Self-Practicing (A-4)

**Figure 13:**
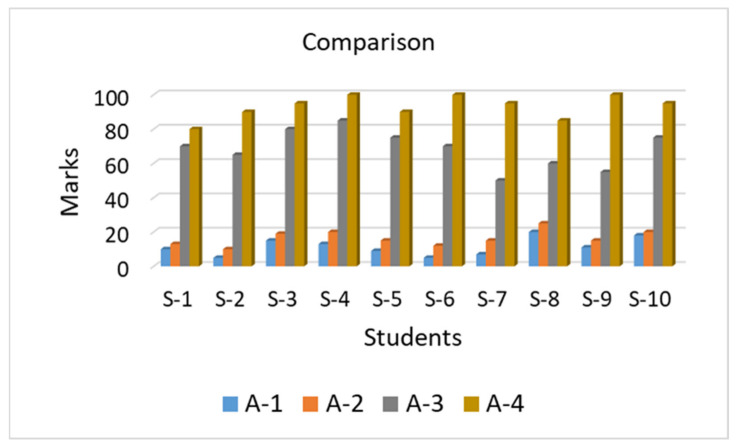
Results comparison of different activities test.

In this session, each student sat in front of a desktop setup individually and played the VR-based game with the help of a keyboard and mouse, with scenes of their own choice. The whole game had to take 10 min, which was already programmed into our system, while the student’s only choice was the environment and avatar selection from predefined options. During their self-practicing, the students were trained enough to be focused on the various interesting game scenes and to follow the game rules as well. They were playing the game in a serious mood, running around to collect butterflies and obtain higher scores. After that, all the students were gathered in a classroom and finally, the last evaluation test was taken, the results of which are shown in Figure 12d. Before the activity, the students did not know about traffic rules and regulations. During the activity when they were becoming used to with the Kinect, they enjoyed performing the VR training and practicing. After the activity, they became familiar with all the road signs, traffic light rules, and road crossing techniques. The students scored good marks by playing the butterfly collecting game. Figure 13 shows a comparative analysis of the results from the students performing a different type of training and learning session. A comparative analysis of the results of different tests based on different training and learning activities shows the high variance in the results. In Figure 13, the students’ names are identified as S-1, S-2, up to the last student that was S-10. The highest available marks are 100 for this test; as is clear from the above results, the students had no basic knowledge about the road safety measures. In the first test, Test-1, that was taken before starting the training sessions, the students scored very low marks. The average results of the participants were 11.3 out of 100 marks. These results show that the students completely lacked road safety knowledge. In the second test, Test-2, when the students were trained with the traditional methods of training and learning, that is, paper and pencil-based, as well as videos and charts, their knowledge was improved to some extent, but they were not trained properly. The average results increased up to 16.4 in the second test. Next, the students were trained and they explored the VE, and the average results of the students increased up to 93 in the final Test-4. 

### 4.5. Children Feedback

The children felt excited during the experiments. They were very much satisfied with the behavior of the avatar pedestrian in the game. Additionally, their response was fascinating. At the start, they enjoyed running through the VE and the traffic alerts. Some of them were getting hit by cars in the beginning, while others were dancing in front of the Kinect sensor. After a while, they got used to the Kinect and started learning the traffic rules of road crossings, as well as other signs. They enjoyed collecting butterflies and the scoring system in the game. All of them tried their best to get high marks, which was only possible if they followed the traffic rules. Some children felt that the cars were real, while others admired the realistic behavior of the avatar and other agents and wanted to walk along with them. After the initial training, during the testing phase, children made minor mistakes. After practicing, most students understood the road signs and demonstrated skills to cross the roads safely, abide by the traffic rules.

### 4.6. Teachers and Parent’s Feedback

The feedback from the parents was very good; they welcomed the virtual city environment and found the response of traffic realistic. They highly appreciated the traffic light system and road symbols and emphasized that these types of activities are beneficial for children, especially at this age. They did not find any drawbacks, but rather found the VRBTS helpful, and especially appreciated the gaming part in which the knowledge gained by children during the training part was provided in an interesting manner. They said that after these activities, they felt more comfortable letting their children go to the market on their own. 

The response from teachers was also positive, as they appreciated the idea and found it motivating and educating. They further added that with the help of this system, not only do children learn faster than traditional teaching, but also emphasized that this will help in reducing the mishaps rate of the children during road crossing. They were fully satisfied with this immersive training procedure and highly recommended this type of activity for children in schools. In Table 2, we have a list of some quotes about our VR training application from parents and teachers that are translated from the regional language into English.

## 5. Conclusions and Future Work

At an early age, road safety training and education are very important for children. Road safety training in a real environment is very challenging and difficult task, and therefore, we need an alternative safe environment for children’ training purpose. To this end, the results of our preliminary study suggest that VEs in combination with a Kinect sensor can make an adequate contribution to educational methodologies, particularly for children. The VE with the assistance of the Kinect sensor described in this paper makes “learning” more entertaining and children consider it as a source of fun with pleasant experiences. Voluntary and involuntary actions are easily trained without any risk of injury. On the other hand, teachers can easily present and assess early children development and reaction times. Simulations and similar strategies are usually used for researching real-world problems and events. Through this, a student can gain new skills and experiences much more easily and reliably than ever before. At the same time, VE is suitable for developing students’ imagination, communicative skills, and creativity, not to mention the bonus of their learning. The main advantage of this research is that it makes the learning and training process interesting and full of entertainment. The main issue with children is that they lack interest in traditional learning and training processes, but with the help of VR and the interaction of the user, they become very attracted toward learning. The children enjoy and entertain themselves while training and learning effectively. The activity results presented in our research show the positive impact of the proposed system on children’s learning. On the other hand, the limitation of our study is that it involved a significantly small number of students due to the COVID-19 pandemic situation. Similarly, the age and class difference between participants was also large. 

The VE presented in this paper can be further enhanced in different directions. Firstly, additional training scenarios can be added, which may include more complex tasks such as riding a bicycle, skating, safely crossing a railroad with personalized contents selection abilities, and many more. Secondly, a more robust grading system can be incorporated, considering the level completion time, comparing results from different students and including audio features for the better involvement of the students. It can also be implemented in healthcare centers for children’s learning and entertainment. Thirdly, additional gestures may be included to provide a more fluid and natural user experience. Next, a specific city or town can be designed with a traffic system similar to the place where children live, and a large number of students can be involved for the generalization of the results. It will be more realistic and easier for students of the same town or city to practice in their hometown or city. Finally, the experience of learning can be boosted to a new level by introducing the features of virtual car driving and tourism to our framework, whereby children can gain real-world experience without any risks of accidents.

## Figures and Tables

**Table 1:** Comparing different setups of our proposed system.

Immersive Setup	Living Room Setup	Desktop Setup
In this setup, we have three large displays arranged in an arc with a Kinect sensor for user interaction with the application.	In this setup, we have a single display along with a multimedia projector with a Kinect sensor attached for user interaction.	In a simple desktop setup, we have a simple keyboard and a mouse for user interaction with a single desktop display.
Immersive setup is comparatively more realistic, it has visual 180 degrees.	The living room setup has a single flat projector display that is comparatively less realistic than the immersive setup.	In this setup, a user is not interacting directly with the VE, and the avatar is controlled with a keyboard and mouse.
A comparatively large space is required for this setup.	For the projection of display, some space is required.	Easy to setup on a simple table.
Immersive setup is only supported by GPU.	It does not require GPU, can be supported by a simple CPU system.	It is a simple CPU setup.
Installation is comparatively harder than the other two setups.	Can be easily installed.	Easy installation.
The immersive setup has higher costs.	It has comparatively lower costs than the immersive setup.	It has a lowest cost.
Immersive setup is not easily moveable from one position to another.	The mobility of the living room setup is easy as compared to the immersive setup.	It is easy to move from one space to another.
This setup is particularly used for training purposes on a large scale, e.g., a whole class.	This setup can also be used as a training system.	This setup is not used for training purposes. It is for the gaming portion of the system.

**Table 2:** Parents’ and teachers’ quotes about VRBTS.

Parents’ Quotes	Teachers’ Quotes
I am happy to see my child involved in learning and at the same time having fun.	The technology involved in such activities is best and helpful for wellbeing.
This application made it possible for the children to see the virtual world, the roads, and the vehicles from different angles that surprise the students.	This setup is quite realistic, our students are enjoying playing, and learning as well.
The government should employ and implement similar strategies in kindergarten so that they can practice road crossing virtually before going out to the actual roads.	Normally, we must take a lot of measures when children cross the roads, such as lining them up and providing them with a teacher while crossing the road. With this application, once we are sure about the learning potentials, we can leave them on their own while crossing the road.
The application is good, students can be trained with their computers by playing games like this.	Good job, your team must visit all the schools in this city and perform this type of activity.
I like the game, my children are playing safely on the road, running on the road, and they are learning (with a smile).	The skills of our students will be greatly improved with these types of activities.
This way of training and practicing has encouraged our children’s creativity.	I liked the randomization strategy of moving cars, it seems so realistic, and the children will be prepared for any type of scenario.
